# Effect of Temperature on Anti-Corrosive Properties of Diamond-Like Carbon Coating on S355 Steel

**DOI:** 10.3390/ma12101659

**Published:** 2019-05-22

**Authors:** Mieczyslaw Scendo, Katarzyna Staszewska-Samson

**Affiliations:** Institute of Chemistry, Jan Kochanowski University in Kielce, Swietokrzyska 15G, PL-25406 Kielce, Poland; staszewska@ujk.edu.pl

**Keywords:** S355 steel, diamond-like carbon (DLC), coating, corrosion, microhardness (HV), alkaline environment

## Abstract

Influence of temperature on the anti-corrosive properties of a diamond-like carbon (DLC) coating, produced using plasma-enhanced chemical vapor deposition (PECVD) on the S355 steel substrate (S355/DLC), was investigated. Corrosion test of the materials were carried out using the electrochemical method. The corrosive environment was an alkaline solution of sodium chloride. The heat treatment of the materials was carried out in air atmosphere, at 400 and 800 °C. It was demonstrated that the DLC coating effectively protected the S355 steel surface from coming into contact with an aggressive corrosive environment. It was found, based on a corrosion test after a heat treatment at 400 °C, that the anti-corrosive properties of the DLC coating did not undergo significant changes. Due to the changes in the surface structure of S355/DLC, the microhardness (HV) of the DLC layer increased. However, after a heat treatment at 800 °C, the carbon coating on the S355 steel surface was destroyed and, thus, lost its protective effect on the substrate.

## 1. Introduction

**Figure 1:**
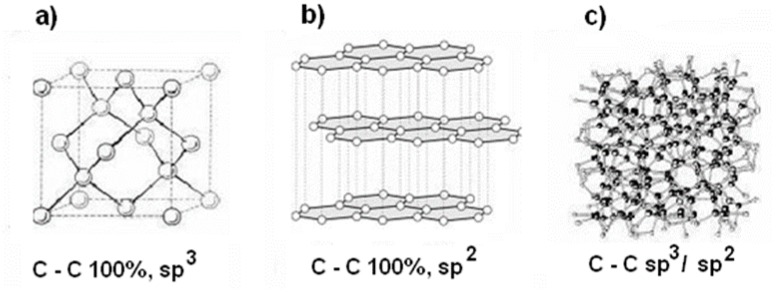
Models of the crystal structure; (**a**) diamond, (**b**) graphite, and (**c**) diamond-like carbon (DLC) coating.

Diamond-like carbon (DLC) is a meta stable form of amorphous carbon with mainly *sp*^3^ hybridization. The *sp*^3^ configuration of DLC confers many beneficial properties on it, such as hardness, chemical and electrochemical inertness, and wide band-gap. Moreover, the DLC consists of cluster of 3-coordinated *sp*^2^ carbon embedded in *sp*^3^ bonded matrix. The *sp*^2^ regions are found to control the electronic properties, such as the band gap, while the *sp*^3^ regions are found to control the mechanical properties, such as rigidity, hardness, fracture toughness and tribological properties such as wear, friction, etc. [1,2,3]. The models of the crystal structure; diamond, graphite, and diamond-like carbon (DLC) coating are shown in Figure 1.

The performances of the DLC films are highly dependent on the atomic C–C bonding (measured by *sp*^3^/*sp*^2^ ratio), which are in turn largely rely on the deposition methods, as well as the deposition parameters. The DLC films can offer a good protection to the metallic substrates, from corrosion. However, the corrosion performance of the DLC coating is closely related to its microstructure or the existence of defects. Porosity is one of the most important parameters in assessing the effectiveness of the corrosion protection of DLC coatings. It is now known that nanoporosity increases with prolonged immersion time. Moreover, the corrosion resistance of ultra-thin DLC films increases substantially with immersion time in an electrolytic solution, due to the filling of pores with a passivating material that prevents the access of the electrolyte to the substrate. On the other hand, the corrosion resistance of the DLC films is increased if the thickness of the diamond-like carbon coating increases [4]. However, the adhesion strength of the DLC films influences the corrosion protection of the films, i.e., higher the adhesion strength of a film, better the corrosion resistance. A sufficient adhesion of the DLC film to its substrate can reduce the undermining effect from corrosive media, and, thus, enhance the corrosion resistance of the film [5,6,7,8]. The structure and properties of DLC films are substantially affected by using deposition methods as well as deposition conditions, such as substrate bias and temperature, gas pressure, dopant, and doping level [9]. However, the substrate temperature and substrate bias are the two major parameters which determine the mobility, as well as the local density of the depositing particles on the substrate and, thereby, the physical properties of the films. Many methods have been developed for producing DLC films, for example, ion beam evaporation, magnenetron sputtering deposition (MSD), plasma immersion ion implantation and deposition (PIII-D), pulse filtered cathodic vacuum arc source technology (FCVA), and pulse laser deposition (PLD) [10]. Moreover, deposition techniques such as Plasma Enhanced Chemical Vapour Deposition (PECVD) can affect the corrosion resistance of DLC films [3]. The PECVD is a new type of technique for deposition of insulating carbon films on conducting, as well as insulating substrates. PECVD techniques are based on glow discharge processes, applying hydrocarbon gases like acetylene (C_2_H_2_) and negatively-biased substrates working at radio frequencies. It is worth noting that DLC coatings can be deposited on the surface of the substrate at low temperatures (<200 °C), thus, elements sensitive to high temperatures are not deformed. The establishment of a relationship between the deposition factors and chemical bonding properties is of great importance to the application of DLC coatings. All DLC coatings have got multiple bonds and varying hydrogen content, within the coating. Depositing a film that combines the proper amount of hydrogen, as well as the proper ratio of *sp*^2^ to *sp*^3^ bonding, and the best underlayer for the application is the key to providing customers with a low friction, a high level of wear resistance, ductility, and a proper adhesion and load carrying capability. At room temperature, DLC films are chemically inert in the entire pH range of the environment. The a-C:H has been shown to demonstrate biocompatibility and has been shown to be effective as a diffusion barrier, including as biomedical implants. So diamond-like carbon is the best material for corrosion protection of materials [1]. The amorphous hydrogenated carbon films deposited on steel, as a protective layer, was reported by Mansano et al. [2]. The combination of steel substrates and amorphous hydrogenated carbon films was used for various applications, including cutting tools and blades [11], as well as automotive parts, such as piston rings [12] and fuel injectors [13]. The anti-corrosive properties of DLC coatings are modified by doping various elements such as, iron [14], nitrogen [15], silicon [16], and titanium [17]. It was found that doping elements changes the structure of DLC coating, surface roughness, and wear resistance. Moreover, it turned out that metallic dopants result in the formation of carbides, which significantly change the mechanical and electrochemical properties of the diamond-like carbon coating. On the other hand, doping nitrogen into DLC gives rise to the transformation of *sp*^2^ to *sp*^3^ carbon hybridization [18]. Furthermore, for non-metallic elements, incorporation of silicon to the DLC coating decreases internal stress and improves tribological behaviour [19]. However, an amorphous hydrogenated carbon (a-C:H) DLC film offer an excellent corrosion protection for the substrates, which are chemically inert to the environment. Therefore, the authors [20] describe the influence of a-C:H on the electrochemical corrosion effect of 4H13 stainless steel (SS), in acidic chloride solution. It has been found that the corrosion rate of SS with a DLC coating, decreased about eight times, in relation to the uncoated surface of stainless steel. The DLC coatings in a low temperature range (up to 100 °C), effectively protect the substrate against corrosion. So far, we have not found in the literature any information on the anti-corrosion properties of DLC coating in an alkaline corrosive environment, before and after heat treatment, at increased and high temperatures.

The aim of this work was to investigate the influence of temperature (400 and 800 °C) on the anti-corrosive properties of diamond-like carbon (DLC) coating on the S355 steel substrate (S355/DLC). Corrosive tests of the investigated materials were carried out in an alkaline chloride environment, using the electrochemical method. The results could be used to determine the corrosion mechanism of the tested materials in a corrosive environment.

## 2. Materials and Methods

The S355 steel was used as the substrate for the diamond-like carbon coating. The chemical composition of the steel was as follows (wt %): C: 0.2; Mn: 1.5; Si: 0.20; P: maximum 0.04; Cr: maximum 0.3; Ni: maximum 0.3; Al: maximum 0.02; Cu: maximum 0.03; and the rest was iron, which is classified as a steel alloy with increased mechanical strength. The surface of the steel substrate was polished with a stream of sand.

**Figure 2:**
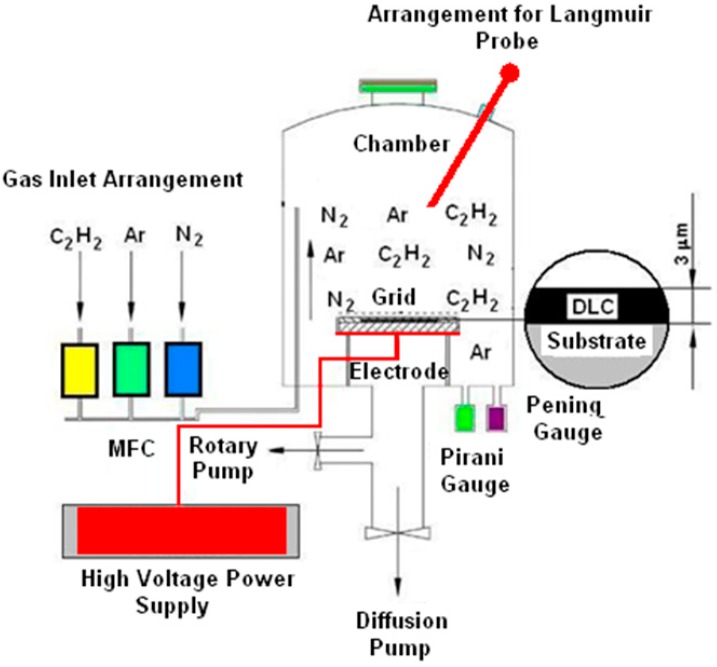
Diagram of equipment for the production of diamond-like carbon coating using the plasma-enhanced chemical vapor deposition (PECVD) method.

The diamond-like carbon (DLC) coating with a thickness of approximately 3 μm on the steel substrate was covered, by using the plasma-enhanced chemical vapor deposition (PECVD) method. The diagram of equipment for the production of DLC coating using the PECVD method, is shown in Figure 2 [3].

In terms of energy, the PECVD deposition process is very beneficial because the plasma requires lesser energy, compared to the heating methods, including the CVD method. The special feature of this device is that only very pure acetylene (C_2_H_2_) must be used for the production of the DLC coatings. An advantage of this system is that there is no need of radio frequency (RF) generator or a pulsed DC supply. Deposition takes place only by using a voltage DC power supply. The chamber for deposition of the DLC film is made of stainless steel, with a diameter of 400 mm and a height of 570 mm. The base pressure, up to 7 × 10^−5^ mbar, is obtained by using a combination of diffusion and rotary pumps. The operating pressure of the system is maintained at 4 × 10^−2^ mbar, by using the gases. Before deposition, the substrates are cleaned using Ar^+^ ions sputtering, using a 1500 DC voltage. Deposition is carried out at a 1000 DC volt, using an acetylene precursor. The carbon layer is deposited by adsorption of hydrocarbon free radicals and formation of chemical bonds, and thus, it is possible to obtain hard, evenly distributed, and well-adhered DLC layers to the substrate. In this case, the amorphous hydrogenated carbon deposition was continued, until the final coating thickness reached about 3 μm on the substrate.

**Figure 3:**
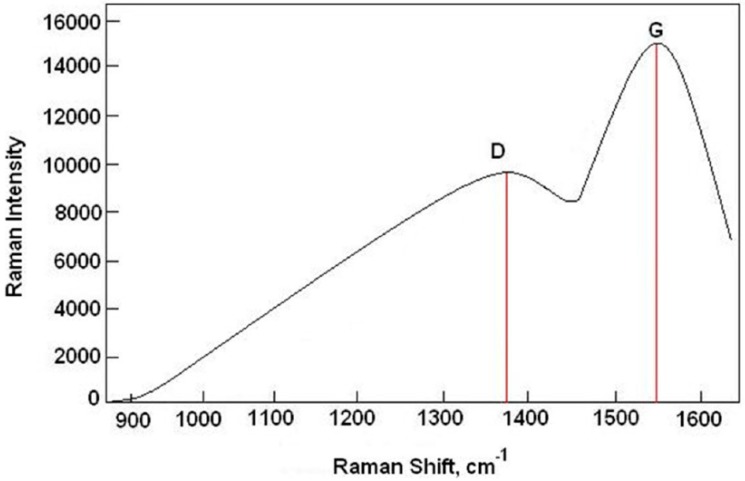
Raman spectrum for the diamond-like carbon coating applied to the surface of S355: D—diamond structure and G—graphite structure.

The best tool for the structure analysis and distinguishing type of bonds in the DLC coatings is Raman spectroscopy (Jobin Yvon T 64000, Palaiseau, France). Figure 3 shows the Raman spectrum of the diamond-like carbon coating.

As it can be seen, peak *D* is located at 1380 cm^−1^. This confirms that the coating has a diamond like structure and a *sp*^3^ configuration. In this configuration, 4 carbon electrons in the valence layer makes a strong σ bond, with the electrons of the adjacent atom. In Figure 3, the peak *G* was at 1550 cm^−1^. This corresponded to the planar graphite in the coating with a *sp*^2^ configuration. In this state, three electrons of the valence layer, formed an σ bond with the electrons of the adjacent atom, and the fourth electron formed a *π* bond which had a weaker strength. The combination of a diamond-like structure and a graphite-like structure caused the desired properties in this coating. Dense diamond like structures played an important role in increasing the yield strength, adhesion, and in improving the chemical and corrosion properties. Moreover, it was found that the ratio of phase *D* (*sp*^3^) to *G* (*sp*^2^) was approximately 0.65, which meant that a good quality diamond-like carbon coating was obtained on the surface of the S355 steel, by using the PECVD method.

### 2.1. Heat Treatment

For the heat treatment of the S355 steel, without and with the DLC coating, an electric chamber furnace type FCF 2.5 HM (CZYLOK, Jastrzebie Zdroj, Poland) was used. The heating process of the specimens was carried out in accordance with the previously edited program. The measuring cycle was one day. The measurement temperature was selected experimentally. The materials were processed in an atmosphere of hot air.

### 2.2. Corrosion Test

All electrochemical measurements (corrosion tests) for the investigated materials were carried out in a conventional thermostated three-electrode cell using the potentiostat/galvanostat PGSTAT 128N (AutoLab, Amsterdam, The Netherlands) piloted by the NOVA 1.7 software.

The working electrode (a stationary) was made of S355 steel, with and without DLC film. All test samples had dimensions of 30 mm × 10 mm × 5 mm, and were fixed in a special holder. The electrical contact of the working electrode with the solution, was 1 cm^2^. The S355 steel surface exposed to the electrolyte was polished with emery papers of grades 150 to 2500 to gain a mirror-like plane. The electrode with the DLC coating was not polished. All working electrodes were cleaned ultrasonically in acetone, ethanol, and distilled water, for 10 min, and were dried under a cold air stream, at room temperature. Subsequently, the working electrode was immediately immersed in the test solution.

The saturated calomel electrode (SCE) was used as the reference. It was connected with the solution using a Luggin capillary. The capillary tip was placed about 3 mm in relation to surface of working electrode.

The counter electrode (10 cm^2^) was made of platinum foil (99.99% Pt).

The potentiodynamic polarization curves were recorded in the alkaline corrosive medium, which was composed of 1.2 M Cl^−^ and 0.2 M OH^−^ (pH 11.5). The following reagents were used for preparing the solutions—sodium chloride (NaCl) CHEMPUR, and sodium hydroxide (NaOH) POCH. The solvent used was three times distilled water. The solutions were not deoxygenated.

Potentiodynamic polarization curves were used to designate the corrosion potential (*E*_corr_) and corrosion current density (*j*_corr_). In order to determine the corrosion parameters, and polarization resistance of the tested materials, the Tafel method was used. More information about the Tafel method can be found in our publications [20,21,22,23].

The electrochemical corrosion rate of the materials were appointed, using the following equation:

(1) $$\nu_{e}=3.268\times \frac{j_{corr}\;M}{n\rho },$$

where *j_corr_* is the corrosion current density, *M* is the molecular weight of the reacting substrate, *n* is the number of electrons exchanged, and *ρ* is the density of materials.

All measurements were carried out at a temperature of 25 ± 0.5 °C, which were maintained using an air thermostat. The experiment was started after 30 min of immersion of the electrode in the testing solution, which was not mixed, and not deoxidized.

### 2.3. Other Measurements

The microhardness of the tested materials was measured using the Vickers method (HV), using the Falcon 500 hardness tester, the INNOVATEST company (Maastricht, The Netherlands). An indenter was used in the form of a diamond pyramid with a square base, and an angle between the opposite walls which equaled to 136°; with a load varying from 0.02 to 20 N. The depth of indentation was about 2 μm.

The X-ray diffraction (XRD, HZG4, Carl Zeiss Jena, Oberkochen, Germany) was used to characterize and analyze the specimens after the heat treatment, the monochromatic Cu, Kα radiation was 1.5407 Å at 30 kV, and 15 mA with a step scan mode, at intervals of 0.05° in 2θ.

The surface structure of the tested materials was observed using a scanning electron microscope (SEM) Joel (Tokyo, Japan), type JSM-5400. The accelerating voltage of SEM was 20 kV.

## 3. Results and Discussion

### 3.1. Corrosion Test

**Figure 4:**
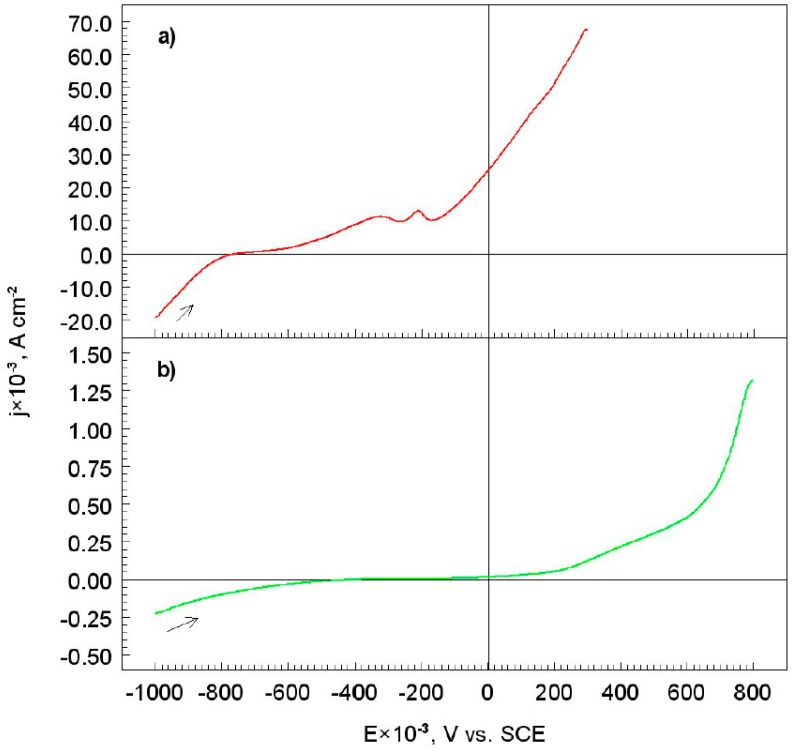
Potentiodynamic polarization curves for: (**a**) S355 steel and (**b**) S355 steel with DLC coating. The solution contained 1.2 M Cl^−^ and 0.2 M OH^−^, dE/dt 1 mV s^−1^, at a temperature of 25 °C.

The corrosion test was conducted using potentiodynamic polarization curves (*LSV*) for the S355 steel, with and without a diamond-like carbon (S355/DLC) coating, in alkaline chloride solutions. All measurements were carried out under a potential range from −1000 to +800 mV, compared to the SCE, whereas the potential change rate was 1 mV s^−1^, Figure 4.

Based on the potentiodynamic polarization curve (Figure 4a), corrosion mechanism of the S355 steel in the alkaline chloride environment was proposed.

The cathodic reaction was as follows:

O_2_ + 2 H_2_O + 4 e^−^ → 4 OH^−^. (2)

The anodic branch for iron showed a short active–passive region (Figure 4a). The active region was due to the dissolution of iron as indicated by the reaction:

Fe → Fe^2+^ + 2 e^−^. (3)

The passive region started from the potential of −0.325 V, compared to the SCE, at which the current density reached its maximum value, i.e., 8 × 10^−3^ A cm^−2^. The current then decreased to show a narrow passivating range of iron in the tested solution, according to the reaction:

Fe + ½ O_2_ + H_2_O → Fe(OH)_2_. (4)

This layer of ferrous hydroxide that had formed reacted further in the presence of excess oxygen in the solution, to build up the final corrosion product (magnetite), according to:

3 Fe(OH)_2_ + ½ O_2_ → Fe_3_O_4_ + 3 H_2_O. (5)

Increasing the potential in the more positive direction resulted in the breakdown of the formed passive film. This was indicated by the rapid increase of current of iron on the curve (Figure 4a). Moreover, in a saline medium, the adsorption of chloride ion on the iron surface developed an intermediate complex that led to the dissolution of the passive film, i.e., the nucleation for the pitting corrosion. However, a peak was observed on the anode curve for the −0.315 V, compared to the SCE electrode potential. Therefore, the following reactions might have elucidated this behavior:

Fe(OH)_2_ + Cl^−^ → Fe(OHCl) + OH^−^, (6)

Fe(OHCl) + n Cl^−^ → [Fe(OH)Cl_(n+1)_]^−n^_ads_, (7)

Fe(OH)_3_ + Cl^−^ → Fe(OH)_2_Cl + OH^−^, (8)

Fe(OH)_2_Cl + n Cl^−^ → [Fe(OH)_2_Cl_(n+1)_]^−n^_ads_. (9)

However, for a more positive electrode potential, the adsorbed products were dissolved according to the reactions:

[Fe(OH)Cl_(n+1)_]^−n^_ads_ + H^+^ → Fe^2+^ + H_2_O + (n + 1) Cl^−^, (10)

[Fe(OH)_2_Cl_(n+1)_]^−n^_ads_ + 2 H^+^ → Fe^3+^ + 2 H_2_O + (n + 1) Cl^−^. (11)

Therefore, a rapid increase in the current density was observed, which resulted from the dissolution of iron in a corrosive environment. A similar mechanism of corrosion of steel, in alkaline chloride solution (Equations (2)–(11)), has been proposed by the authors of [24,25].

The potentiodynamic polarization curve for the S355 steel coated with DLC coating is shown in Figure 4b. It is clearly visible that the cathodic and anode current density decreased drastically, compared to S355 electrode, without any DLC coating. Thus, the S355/DLC layer was tight and adhered well to the surface of the steel electrode, preventing it from oxidation, in the aggressive chloride environment.

Potentiodynamic polarization curves (Figure 4) were used to determine the parameters of electrochemical corrosion of the S355 steel, with and without a diamond-like carbon coating, (listed in Table 1, along with the polarization resistance of the electrodes).

The corrosion potential for the S355/DLC shifted considerably (about 0.350 V) towards positive values, compared to the corrosion potential of the substrate. This meant that the DLC coating had an anti-corrosive effect on the S355 steel. However, the value of corrosion current density for the substrate was more than seventy times higher, compared to the S355/DLC (Table 1). The DLC coating protected the substrate from coming in contact with an aggressive chloride environment. Moreover, the values of the cathodic and anodic Tafel slopes (bc and ba) for the tested materials differed significantly (Table 1). Therefore, it could be concluded that the mechanism of the corrosion process of the S355 surface, without a diamond-like carbon coating was different (Equations (2)–(11)). On the other hand, the value of the polarization resistance for the S355/DLC was higher than for the substrate (Table 1). The DLC layer on the surface of the S355 steel, behaved in a manner similar to a typical insulator, therefore, in the experimental conditions it was difficult to replace the electrical charge between the electrode and the electrolyte.

### 3.2. Thermogravimetric Measurement

**Figure 5:**
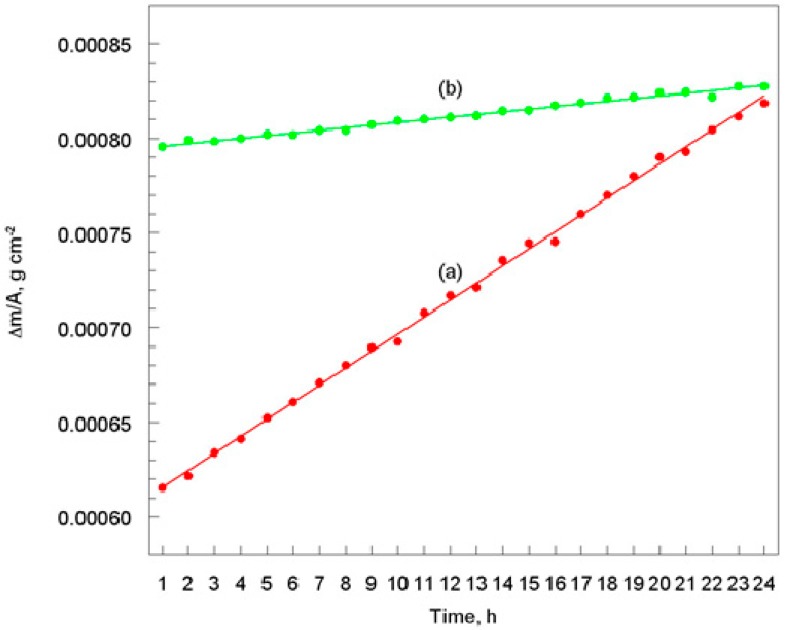
Influence of exposure time on mass change, during heat treatment in air atmosphere: (**a**) S355 steel and (**b**) S355 steel with DLC coating. Exposure temperature of 400 °C.

To investigate the effect of temperature on the anti-corrosion properties of the diamond-like carbon coating, the S355/DLC samples were heat-treated in an air atmosphere. Moreover, for comparison, similar measurements were made for the S355 steel. The processing temperature was set at the so-called *critical temperature* [20], which was determined on the basis of a separate measurement. It was found that the critical temperature was about 430 °C, for the S355 steel, while for the S355/DLC, it was about 780 °C. The thermogravimetric measurements consisted in registering the weight gain (*W = Δm/A*) of the tested materials as a function of time, in this case for a temperature of 400 °C, Figure 5.

It was found that the process of chemical corrosion (oxidation) of the materials, proceeded according to the linear law: *W* = *k t* + *C*, which was characteristic for the metals whose oxide surface was porous [26]. The kinetic equation that described the oxidation process of S355 steel was:

*W* = 8.97 *t* + 6.07 × 10^−4^. (12)

However, in the case of 355 steel coated with DLC, the kinetic equation could be written as:

*W* = 1.39 *t* + 7.95 × 10^−4^. (13)

The surface of the substrate under hot air conditions (400 °C) was covered with a thick porous oxide layer, which did not create an effective obstacle in the access of the oxidant to the metal surface. Therefore, its mass significantly increased (over 30%), compared to the mass of the same specimen before the experiment (Figure 5a). However, no significant surface changes were observed for the S355/DLC. In this case, after the measurement, the mass of the test specimen changed slightly (about 3%; Figure 5b). In addition, the diamond-like carbon coating still adhered well to the surface of the substrate. It could be assumed that the DLC layer covering of the S355 surface did not significantly lose its anti-corrosive properties after being exposed to an atmosphere of hot air heated to 400 °C.

Similar thermogravimetric measurements were also made for the materials S355 and S355/DLC, at 800 °C (results are not quoted). Due to the high ambient temperature, the diamond-like carbon coating was clearly damaged, partially fractured, and in some places, was separated from the surface of the S355 substrate. Moreover, some cracks in the DLC coating were filled with iron oxides. Thus, the S355/DLC layer probably lost its protective properties to the substrate.

**Figure 6:**
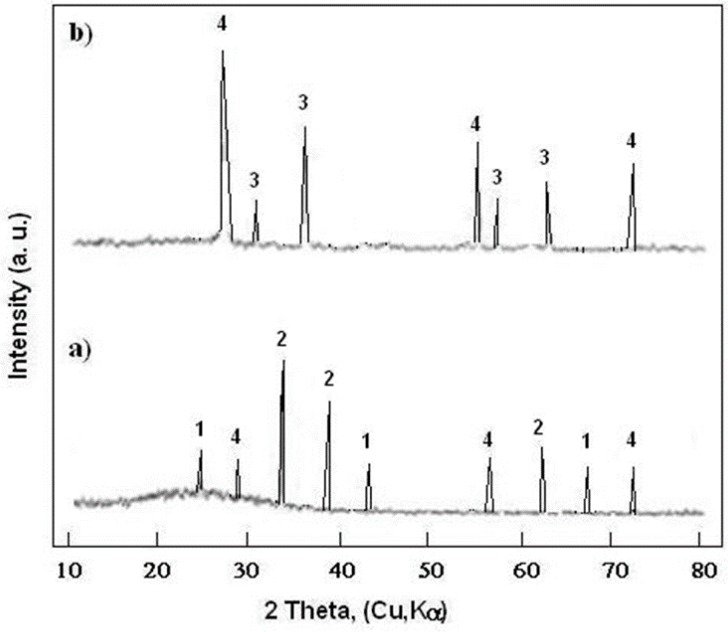
X-ray diffraction of the S355 steel surface after heat treatment in air atmosphere at: (**a**) 400 °C and (**b**) 800 °C. 1—FeO, 2—Fe_2_O_3_, 3—Fe_3_O_4_, and 4—graphite.

The X-ray diffraction results of the S355 steel, after heat treatment at 400 and 800 °C, are presented in Figure 6.

It was found (Figure 6a) that after the heat treatment at 400 °C, the substrate was covered with a layer of FeO and Fe_2_O_3_ oxides:

Fe + ½ O_2_ ↔ FeO, (14)

2 FeO + ½ O_2_ ↔ Fe_2_O_3_. (15)

However, after heat treatment at 800 °C (Figure 6b), the oxide layer of FeO and Fe_2_O_3_ were transformed, according to the reaction:

FeO + Fe_2_O_3_ ↔ Fe_3_O_4_. (16)

Therefore, the surface of the S355 substrate was covered with a thick layer of Fe_3_O_4_.

#### Microhardness of the Materials

The results of microhardness measurements (HV) for the S355 steel, with and without the diamond-like carbon coating, before and after heat treatment at 400 °C, in one day, are listed in Table 2. It was found that the microhardness (HV) of a specimen with a diamond-like carbon coating increased almost four times, compared to the microhardness of the substrate. In addition, after heat treatment, the microhardness of the S355 steel coated with the DLC coating, increased by about 9% compared to the S355/DLC specimen before heat treatment (Table 2).

Thus, as a result of the heat treatment at 400 °C, the surface structure of the S355/DLC specimen changed, which increased the microhardness of the surface.

### 3.3. Corrosion Test after Heat Treatment

In order to investigate the effect of temperature on the anti-corrosion properties of the diamond-like carbon coating on the S355 substrate, the potentiodynamic polarization curves were recorded in an alkaline chloride solution, after the heat treatment of materials in a hot air atmosphere of 400 and 800 °C.

**Figure 7:**
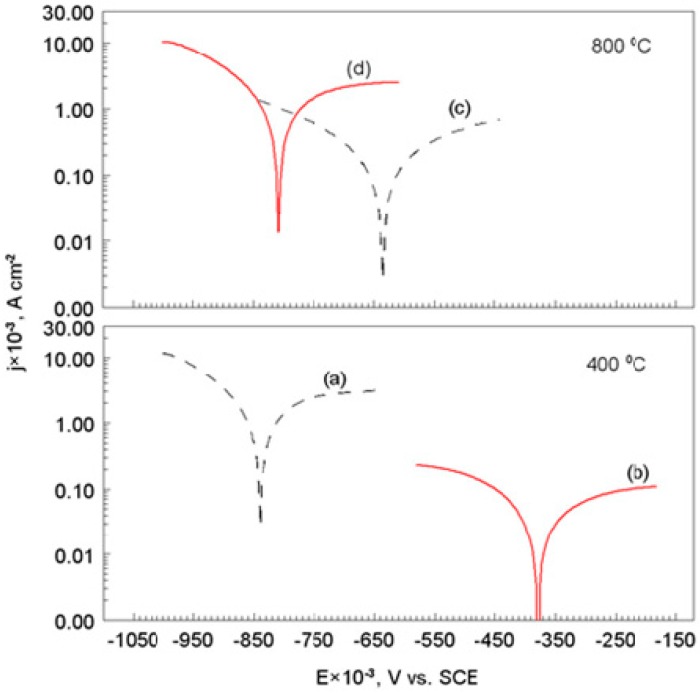
Potentiodynamic polarization curves (semilogarithmic scale) for the S355 steel, with and without DLC coating, after heat treatment in air atmosphere, at 400 and 800 °C: (**a**) S355, (**b**) S355/DLC, (**c**) S355, and (**d**) S355/DLC. Solution contained: 1.2 M Cl^−^, and 0.2 M OH^−^, dE/dt 1 mV s^−1^, temperature 25 °C.

Figure 7a,c show the potentiodynamic polarization curves (semilogarithmic scale) for the S355 steel without, and with DLC coating, after the heat treatment in air atmosphere, respectively, at 400 and 800 °C.

The electrochemical corrosion parameters, and the polarization resistance values for the tested materials, are listed in Table 3. Corrosion potential of the S355/DLC after heat treatment at 400 °C was significantly shifted (about 0.460 V) towards positive values, compared to the corrosion potential of the substrate (coated with a layer of FeO and Fe_2_O_3_, Equations (14) and (15)). Similarly, the corrosion current density, and polarization resistance were clearly decreased in the case of the S355/DLC (Table 3). The results of the corrosion test indicated that the S355/DLC coating, after heat treatment at 400 °C, retained its anti-corrosive properties. Moreover, it was found that S355 steel after heat treatment at 800 °C was covered with a thick layer of mainly Fe_3_O_4_ (Equation (16)), which effectively protected the substrate from coming in contact with aggressive electrolytes, as evidenced by the low value of the corrosion current density (about 0.29 × 10^−3^ A cm^−2^); Table 3. In the case of the S355/DLC coating, corrosion test in the alkaline chloride environment showed a loss of the anti-corrosive properties of the diamond-like carbon coating. The corrosion potential had shifted towards negative values (about 0.170 V), and corrosion current density increased more than five times, compared to the S355 as a substrate material. It is worth noting that the values of the cathodic Tafel slopes (bc) changed for the S355/DLC materials (compared to S355) that had been heat treated at 400 and 800 °C, respectively (Table 3).

This meant that the oxygen reduction mechanism (Equation (2)) on the S355 surface was different than that in the case of S355/DLC. Moreover, the value of the anodic Tafel slopes (ba) had not been determined. As a result of the destruction of the DLC layer on the surface of S355 steel, the exchange of mass and electric charge between the electrode and the electrolyte was facilitated, and thus, the effects of electrochemical corrosion of the substrate in the alkaline chloride environment, was clearly visible. This problem will be discussed later in this work.

#### 3.3.1. Corrosion Rate

Potentiodynamic polarization curves (Figure 4b and Figure 7, curves (b), (d)) were used to calculate the electrochemical corrosion rates (Equation (1)), in an alkaline chloride solution, for the S355 steel with DLC coating, before and after heat treatment in air atmosphere at 400 and 800 °C, Table 4.

The electrochemical corrosion rate of the S355/DLC in the alkaline chloride solution, was very small and amounted to 0.04 mm/year. Thus, the DLC coating very effectively protected the substrate against the effects of the electrochemical corrosion. Heat treatment in air atmosphere at 400 °C of S355/DLC, significantly reduced the anti-corrosive properties of the diamond-like carbon layer. It was found that the corrosion rate of the material increased to 0.69 mm/year (Table 4). It seemed that the thickness of the DLC coating decreased as a result of a uniform oxidation of the surface of the layer. This meant that oxygen could diffuse into the DLC layer, by oxidizing the substrate. A two-fold increase in air temperature to 800 °C, resulted in a significant damage to the surface of the DLC layer, which made the substrate corrosion rate increase by about twenty-six times, compared to the previous rate of the corrosion, i.e., 0.69 mm/year (Table 4). Therefore, under high temperature conditions, the S355/DLC coating did not protect the substrate against chemical corrosion. Under the experimental conditions, the S355 steel surface was covered with a thick oxide layer, predominantly of Fe_3_O_4_ (Equation (16)), which adhered well to the surface of the substrate.

#### 3.3.2. SEM of the Cross-Section

**Figure 8:**
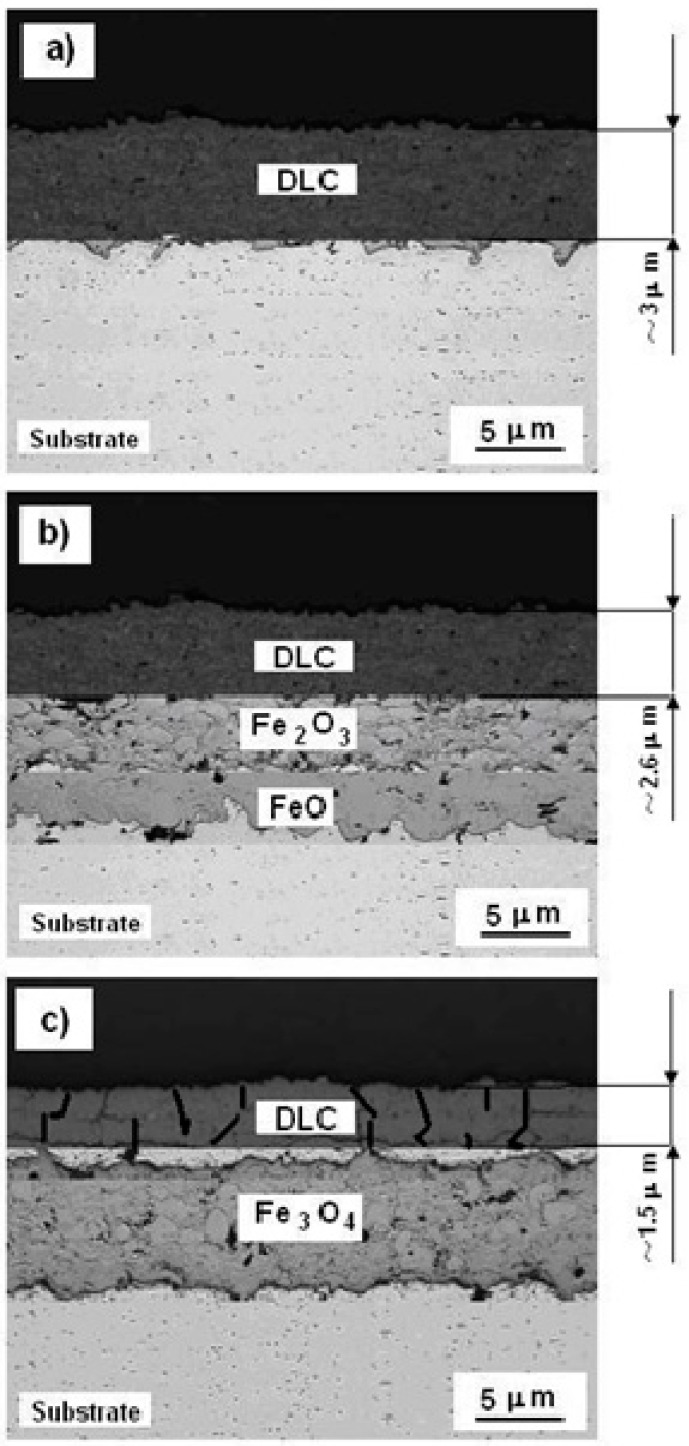
SEM of a cross-section of the S355 steel, with DLC coating, before and after heat treatment in air atmosphere, at temperatures: (**a**) room-temperature, (**b**) 400, and (**c**) 800 °C. The exposure time was about 24 h.

Figure 8 shows the SEM of cross-section of the S355 steel, covered with a diamond-like carbon coating, before and after heat treatment in a hot air environment, which had a temperature of 400 and 800 °C, respectively. The time of exposure was one day. It was clearly visible that the DLC coating with a thickness of about 3 μm, adhered well to the substrate, its structure was finely crystalline, homogeneous, and had no cracks (Figure 8a), which allowed it to properly protect the substrate from coming in contact with aggressive corrosive environment. Heat treatment of S355/DLC material at a temperature of 400 °C, caused the thickness of the DLC coating to decrease, as a result of the uniform oxidation of its surface, in this case, it reached a value of about 2.6 μm. As a result of the diffusion of oxygen through the DLC layer, the substrate was covered with a layer of iron (II) oxide (Figure 8b). The layer of FeO oxide, under these conditions, acted as an electrolyte and electron conductor, and its external surface was a cathode. The layer of FeO oxide under these conditions acted as an electrolyte and electron conductor, and its external surface was a cathode, while on its surface, a reaction occurred:

O_2_ + 4 e^−^ ↔ 2 O^2−^. (17)

Therefore, the anodic reaction was as follows:

Fe ↔ Fe^2+^ + 2 e^−^, (18)

which ran at the metal–oxide interface, and the cathodic Equation (17) at the oxide–environment interface.

It is known that oxide electron conductivity is one order of magnitude higher than its ionic conductivity, therefore, ion diffusion determines the rate of oxidation of the substrate [20], which immediately transforms into the iron(III) oxide in the hot air environment (Equation (15)). Therefore, the substrate was covered with a thick oxide layer of FeO/Fe_2_O_3_; Figure 8b.

On the other hand, an increase in air temperature to 800 °C resulted in a significant destruction of the DLC surface structure (Figure 8c), numerous cracks had appeared, therefore, oxygen diffused easily into the layer, and the substrate was covered with an inhomogeneous, coarse, oxide layer of Fe_3_O_4_ (Equation (16)). In addition, the thickness of the DLC layer was clearly reduced two-folds, to a value of 1.5 μm. Therefore, the DLC coating lost its anti-corrosive properties in the measurement conditions, and oxygen easily penetrated into the material, oxidizing the subsequent layers of the substrate.

## 4. Conclusions

On the basis of the above collected data, the following conclusions could be drawn:

1. The S355 steel surface was covered with diamond-like carbon layer (DLC), using the Plasma-Enhanced Chemical Vapour Deposition (PECVD) method. The ratio of the diamond phase (*sp*^3^) to graphite phase (*sp*^2^) was about 0.65.
2. In the alkaline chloride environment, S355 steel underwent electrochemical corrosion, according to a multi-step mechanism, and the S355 electrode surface was coated with porous iron(III) oxide (magnetite), which adhered well to the substrate.
3. The corrosion test conducted by using the electrochemical method, showed that, the S355/DLC coating was tight and protected the S355 substrate from contacting with an aggressive solution.
4. Thermogravimetric measurements showed that the chemical corrosion process of the tested materials could be described by using the linear law.
5. After heat treatment at 400 °C, the S355 substrate was covered with a layer of FeO/Fe_2_O_3_, and for 800 °C, the substrate was covered with a thick layer of Fe_3_O_4_.
6. It was found that after heat treatment at 400 °C of DLC coating, it partially lost its anti-corrosive properties. As a result of the structural changes, the hardness (HV) of S355/DLC was increased four times, as compared to HV of the substrate.
7. Carbon coating on the S355 steel surface was destroyed after heat treatment at 800 °C and the DLC lost its protective properties. The S355 substrate was covered with an inhomogeneous, coarse oxide layer of Fe_3_O_4_.

## Figures and Tables

**Table 1:** Parameters of electrochemical corrosion, and polarization resistance of the S355 steel electrode, with and without a diamond-like carbon coating, at a temperature of 25 °C.

Materials	E_corr_ V vs. SCE × 10^−3^	j_corr_ A cm^−2^ × 10^−3^	−bc	ba	R_p_ kΩ cm^2^
			V/dec		
S355	−759	0.260	0.080	0.230	99
S355/DLC	−409	0.0034	0.155	---	128

**Table 2:** Microhardness for the S355 steel, with and without the diamond-like carbon coating, before and after heat treatment, in air atmosphere at 400 °C, in 24 h.

Materials	Microhardness					
	HV0.02	HV0.5	HV2.0	HV5.0	HV10.0	HV20.0
S355	263	237	217	172	152	136
Before heat treatment						
S355/DLC	974	878	804	636	562	503
After heat treatment						
S355/DLC	1071	966	884	700	618	550

**Table 3:** Parameters of electrochemical corrosion, and polarization resistance for the S355 steel, with and without the diamond-like carbon coating, after heat treatment in air atmosphere at 400 and 800 °C.

Materials	E_corr_ V vs. SCE × 10^−3^	j_corr_ A cm^−2^ × 10^−3^	−bc	ba	R_p_ kΩ cm^2^
			V/dec		
400 °C					
S355	−838	2.30	0.205	---	0.2
S355/DLC	−378	0.059	0.250	---	7
800 °C					
S355	−634	0.29	0.295	---	1.5
S355/DLC	−806	1.54	0.185	---	0.3

**Table 4:** Electrochemical corrosion rate of the S355 steel with a diamond-like carbon coating, before and after heat treatment in air atmosphere at 400 and 800 °C, the electrolyte contained 1.2 M Cl^−^ and 0.2 M OH^−^, at a temperature of 25 °C.

Materials	υ_e_ mm/year
S355/DLC	0.04
S355/DLC Temperature 400 °C	0.69
S355/DLC Temperature 800 °C	17.90

## References

[1] Zang A., Liu E., Annergren I.F., Tan S.N., Zhang S., Hang P., Gao J. (2002). EIS capacitance diagnosis of nanoporosity effect on the corrosion protection of DLC films. Diam. Relat. Mater..

[2] Mansano R.D., Massi M., Mousinho A.P., Zambom L.S., Nato L.G. (2003). Protective carbon layer for chemical corrosion of stainless steel. Diam. Relat. Mater..

[3] Sharma R., Barhai P.K., Kumari N. (2008). Corrosion resistant behaviour of DLC films. Thin Solid Films.

[4] Khun N.W., Liu E., Zeng X.T. (2009). Corrosion behavior of nitrogen doped diamond-like carbon thin films in NaCl solutions. Corros. Sci..

[5] Liu C.I., Hu D.P., Xu J., Yang D.Z., Qi M. (2006). In vitro electrochemical corrosion behavior of functionally graded diamond-like carbon coatings on biomedical Nitionol alloy. Thin Solid Films.

[6] Azzi M., Paquette M., Szpunar J.A., Klemberg-Sapieha J.E., Martinu L. (2009). Tribocorrosion behaviour DLC-coated 316L stainless steel. Wear.

[7] Zho G.H., Aunc R.E., Espallargas N.E. (2016). Tribocorrosion studies of metallic biomaterials: The effect of plasma nitriding and DLC surface modifications. J. Mech. Behav. Biomed. Mater..

[8] Bueno A.H.S., Solis J., Zhao H., Wang C., Simoes T.A., Bryant M., Neville A. (2018). Tribocorrosion evaluation of hydrogenated and silicon DLC coatings on carbon steel for use in values, pistons and pumps in oil and gas industry. Wear.

[9] Liu E., Kwek H.W. (2008). Electrochemical performance of diamond-like carbon thin films. Thin Solid Films.

[10] Lu Z.G., Chung C.Y. (2008). Electrochemical characterization of diamond like carbon thin films. Diam. Relat. Mater..

[11] Sakon S., Hamada T., Fujimoto S., Umesaki N., Kobayashi A. (2008). Surface modification of electric hair clipper blade for increasing its lifetime. Vacuum.

[12] Tung S.C., Gao H. (2003). Tribological characteristics and surface interaction between piston ring coatings and a blend of energy-conserving oils and ethanol fuels. Wear.

[13] Treutler C.P.O. (2005). Industrial use of plasma-deposited coatings for components of automotive fuel injection systems. Surf. Coat. Technol..

[14] Ray S.C., Pong W.F., Papakonstantinou P. (2016). Iron, nitrogen and silicon doped diamond like carbon (DLC) thin films: A comparative study. Thin Solid Films.

[15] Corona-Gomez J., Shiri S., Mohammadtaheri M., Yang Q. (2017). Adhesion enhancement of DLC on CoCrMo alloy by diamond and nitrogen incorporation for wear resistant applications. Surf. Coat. Technol..

[16] Bociaga D., Sobczyk-Guzenda A., Szymanski W., Jedrzejczak A., Jastrzebska A., Olejnik A., Swiatek L., Jastrzebski K. (2017). Diamond like carbon coatings doped by Si fabricated by a multi-target DC-RF magnetron sputtering method–mechanical properties, chemical analysis and biological evaluation. Vacuum.

[17] Huang L., Yuan J., Li C., Hong D. (2018). Microstructure, tribological and cutting performance of Ti-DLC/α-C:H multilayer film on cemented carbide. Surf. Coat. Technol..

[18] Zeng C., Chen Q., Xu M., Deng S., Lou Y., Wu T. (2017). Enhancement of mechanical, tribological and morphological properties of nitrogenated diamond-like carbon films by gradient nitrogen doping. Diam. Relat. Mater..

[19] Tanaka I., Nakano T., Kousaka H., Hashitomi H. (2017). Tribological behavior of unlubricated sliding between a steel ball and Si-DLC deposited by ultra-high-speed coating employing an MVP method. Surf. Coatings Technol..

[20] Scendo M., Staszewska-Samson K. (2017). Effect of surface modification on corrosion resistance of uncoated and DLC coated stainless steel surface. J. Mater. Eng. Perform..

[21] Scendo M., Radek N., Trela J. (2013). Influence of laser treatment on the corrosive resistance of WC-Cu coating produced by electrospark deposition. Int. J. Electrochem. Sci..

[22] Scendo M., Trela J., Antoszewski B., Kargul T. (2014). Corrosion resistance of the joint of stainless steels in aggressive solution, Innovations in Corros. Mater. Sci..

[23] Scendo M., Trela J., Radek N. (2014). Influence of laser power on the corrosive resistance of WC-Cu coating. Surf. Coat. Technol..

[24] Sherif E.S.M. (2011). Effects of 5-(3-aminophenyl)-tetrazole on the inhibition of unalloyed iron corrosion in aerated 3.5% sodium chloride solutions as a corrosion inhibitor. Mater. Chem. Phys..

[25] Matos L.C., Martins J.I. (2018). Analysis of an Educational cathodic Protection System with a single drainage point: Modeling and experimental validation in aqueous medium. Materials.

[26] Staszewska K., Scendo M. (2016). Mechanism and kinetics oxidation of Inconel 617 and 625 alloys. Tech. Issues.

